# Modified Bluegrass Appliance: A Nonpunitive Therapy for Thumb Sucking in Pediatric Patients—A Case Report with Review of the Literature

**DOI:** 10.1155/2013/537120

**Published:** 2013-05-22

**Authors:** Amish Diwanji, Preet Jain, Jigar Doshi, Prakash Somani, Dhaval Mehta

**Affiliations:** ^1^Department of Pedodontics and Preventive Dentistry, Faculty of Dental Science, Nadiad, Gujarat, India; ^2^Department of Prosthodontics, Pacific Dental College, Udaipur, India; ^3^Department of Orthodontics and Dentofacial Orthopedics, Darshan Dental College, Udaipur, India; ^4^Department of Oral Medicine and Radiology, Karnavati School of Dentistry, Gandhinagar, Gujarat, India

## Abstract

Oral habits in form of digit/thumb sucking are common phenomenon and part of childhood behavior. They are normally associated with oral pleasure, hunger, anxiety, and sometimes psychological disturbances. Chronic practice can cause major orthopedic alterations to the skeletal structures of the oral cavity and lower face. Aversive approaches in form of punitive therapy have been moderately effective. Modified bluegrass appliance is nonpunitive therapy to treat sucking habits. It acts as a habit reversal technique and installs positive reinforcement in children. Modified blue grass appliance proved to be very comfortable to patients and encourages neuromuscular stimulations.

## 1. Introduction

Oral habit is a part of normal development in children. Habits are learned patterns of muscle contraction with complex nature. Oral habits are repetitive act seen commonly from infancy and should finish automatically as age advances. Common repetitive behavior seen in infants is hand or figure sucking [1]. Sucking is one of the most common reflexes seen in infants. It manifests when they are in womb around 29 wk of age [2]. This is the first pattern of behavior observed in infant. Infants and young children may use finger, thumb, pacifiers, or other objects to feel secure and learn the outside world. This is commonly seen when the child is anxious, insecure or surrounded by strangers and in families when they are separated from their parents. Sucking habit induces sleep and hence makes infant and child calm and relaxed [3]. 

Hand sucking is naturally developed in 89% of infants in the second month and increases by first year of life [1]. It is normal up to 2–4 years of age. It becomes a concern when continued for longer time and even seen in mixed dentition phase. This is the first sign for child to manifest future malocclusion or discrepancy during mixed dentition. The prevalence of oral habit has been reported up to 88% and 30% in high school girls and boys, respectively [4]. 34% of prevalence has been reported in other literature [5]. It has been documented that parental education, child's nutrition, and sucking habits are associated with each other [6]. However, higher prevalence rate has been claimed with high stress level among children in recent time [7]. 

When child performs sucking habit in the first year of life, parents can move away his/her thumb smoothly and attract the child to other things. After second year of age, sucking habit should start decreasing and should appear only when child goes to sleep [1]. When it continues in mixed and permanent dentition, it becomes a parental concern, as it is believed to affect growth of maxilla palate leading to skeletal changes, showing side effects on developing occlusion and resulting in malocclusion and constriction of skeletal structures [8, 9]. 

The effect of prolonged sucking habit in children can affect development of occlusion. It may result in anterior open bite, increased over jet, lingual inclination of lower incisor and labial inclination of maxillary anterior, posterior crossbite, deep palate, compensatory tongue thrust, and sometimes speech defect [10, 11]. The changes in dentition depend upon duration and frequency of habit being performed. During active eruption phase of permanent teeth, children who perform sucking habit for longer duration (more than 6 hrs in a day), especially during sleep, tend to develop minor skeletal abnormalities and sever dentoalveolar changes [12].

Historically, correction of habit revolves around direct counseling of child, encouragement to improve self-confidence by rewarding the child, appliance therapy, and, in case of more complex dental changes, orthodontic therapy along with habit breaking appliances [1]. The use of pacifier may cause severe and harmful effects on dentition if used for more than 5-year-old child [13]. Tongue, Cribs, Hay rakes and other sharp points employ an aversive negative stimulus to cease the undesirable oral habits. They are moderately effective and may trigger unexpected behavior sometimes. In 1991, Haskell and Mink introduced Blue grass appliance, also known as habit correction roller which gained universal attention and acceptance [14]. It is userfriendly, nondestructive, easy to wear appliance replacing the common destructive habits. It is useful in avoiding traditional physical barriers of appliance in form of cribs and helping child with positive reinforcement. Later, similar appliance called Lingual Pearl was used as a habit breaking and for multiple clinical applications [15]. Further, Baker modified blue grass appliance with multiple rollers/beads and thus expanding its use from primary to permanent dentition [16].

## 2. Management 

Management of sucking habit depends upon the age. Many questions arise in parents' mind and among pediatricians regarding intervention by specific therapy to break the habit. Counseling by pediatric dentist and pediatrician is important. If habit is stopped by 4-5 years of age, However, when they persists during eruption of permanent teeth child should be motivated to stop the habit [17, 18].

Appliance therapy involves use of either fixed or removable design in form of palatal crib or spurs. It is reminder therapy for child to make the habit unpleasant and difficult to practice. However, it causes difficulty in speech and eating and can cause iatrogenically inflicted wound and make child emotionally disturbed [14, 19]. Here, we present a case of thumb sucking habit where habit was corrected using Bluegrass appliance as a non punitive therapy.

### 2.1. Case Report

**Figure 1:**
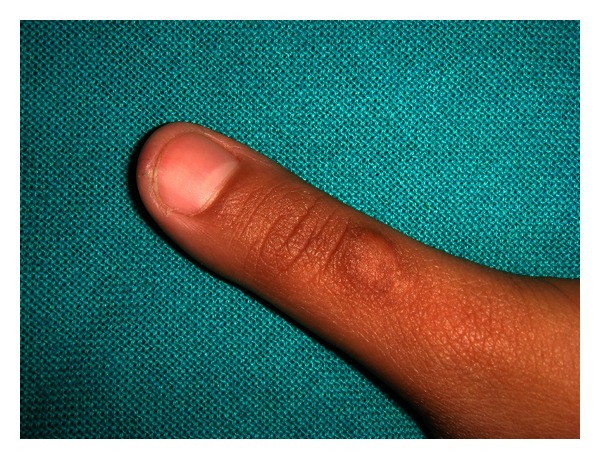
Callous formation was seen over digits of patient.

**Figure 2:**
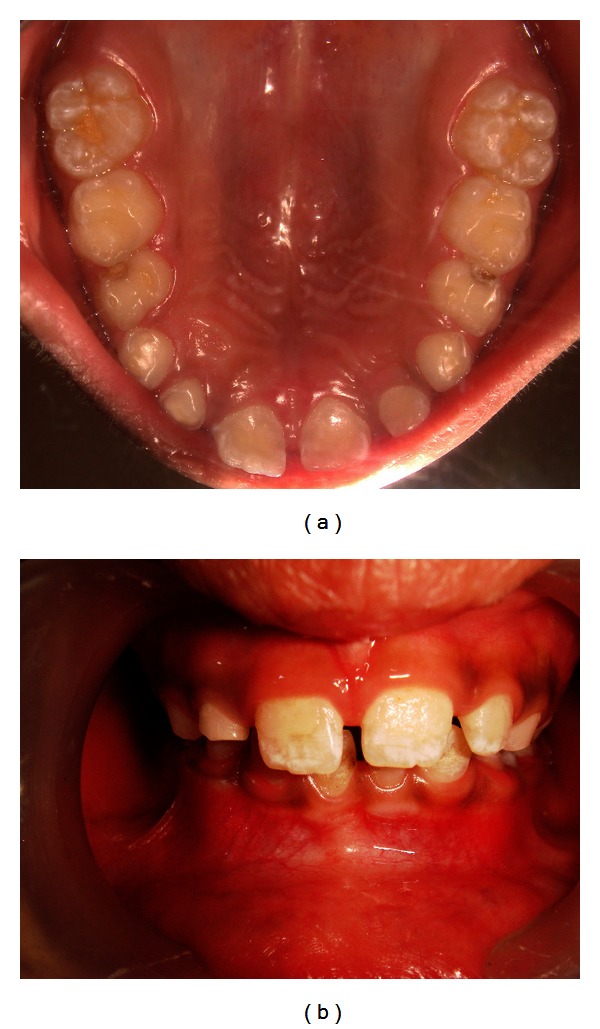
Proclination of teeth and increased overjet and overbite with high palatal vault.

**Figure 3:**
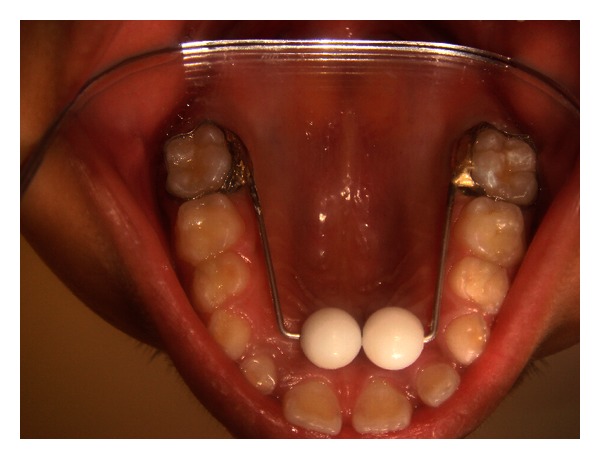
Modified blue grass appliance.

We present a case of an 8-year-old child, gir,l whose parents reported proclination of maxillary teeth and also complained of thumb sucking habit since birth. The patient used to suck her thumb regularly, 8-9 hrs/day, unconsciously in sleep or when idle from the primary dentition period. Callous formation was seen over her digits (Figure 1). The patient reported with proclination of teeth, increased overjet and overbite with high palatal vault (Figures 2(a) and 2(b)). Management was started by counseling the parent and the child regarding the ill effects of digit sucking on the developing dentition during the first visit. On the second visit, the patient was willing to discontinue the habit by treatment. A modified blue grass appliance was planned. Molar bands were fabricated and adapted on maxillary molars. Alginate impression was taken and casts were poured with dental stone over which molar bands were transferred. Stainless steel wire (0.9 mm) was adapted over the palate extending from either side of molars. Acrylic beads were made in laboratory using dental monomer and polymer. Later, beads were inserted into stainless steel wire over palatal rugae area. No contact was established by beads with palatal tissues. The wire was soldered to molar bands by protecting the beads. The appliance was cemented using luting cement (Figure 3). The patient was instructed to roll the bead with tongue whenever she feels like sucking her thumb. The patient was kept on followup every month for checkup. The child was comfortable with the appliance and played by rolling the beads with the tongue. By end of 4 months, callous formation had almost disappeared. Patient was asked to wear appliance for almost 6 months after correction to avoid relapse of the habit. Appliance was removed after the discontinuation of habit. 

## 3. Discussion

Digit sucking is common phenomenon in pediatric age group that reflects the earliest form of habitual manipulation of body. Many questions arise in the minds of general dentist, pediatricians, pediatric dentists and psychiatrists regarding impact of sucking habits on developing dentition. 

When should an attempt be made to break the habit? How does it disturb the child psychologically? In case of major orthopedic alterations to the skeletal structures of the oral cavity and lower face, intervention of orthodontist may require correct malocclusion. Since years, habit breaking appliances in form of palatal cribs, spurs, palatal bars, hay rakes, and cage type appliances have been given to pediatric age group. However, emotional disturbances, difficulty in speech and eating, and iantrogenically self-inflicted wounds can occur with such appliances. Hay rake and cage type appliances tend to get mutilated or destroyed while eating or due to habitual sucking habit. It reminds the child as punitive therapy to cease the habit [19]. Haskell and Mink described that blue grass appliance which is easy to wear, and did not have problems associated with traditional palatal cribs. The design consisted of hexagonal Teflon roller on a cross-palatal wire which was effective to ending the sucking habit in several days [14]. 

In the present study, modified blue grass appliance was used using 3 mm acrylic beads as per recommended by Baker [16]. It encourages neuromuscular stimulations by using multiple beads as per what was the principles of Castillo-Morales [20]. Between 4–6-year-old children can be instructed to play with the beads with the tongue immediately after placement. This allows the child to accept the appliance and learn the neuromuscular activity to normalize the tongue position. When a spinning roller is placed in close proximity to the tip of the tongue, “fascinating” response is quickly implemented due to neuromuscular and sensitive nature of tongue. Since Teflon rollers are not in contact with palatal tissues, children can roll them with their tongues. Within few days, the tongue establishes new nonharmful habit of playing with roller. Hence, this appliance works through counter conditioning response to the original conditioned stimulus for thumb sucking. 

Psychologically, it is acceptable for parents also as they can encourage the child to play with beads instead of instructing the child to cease the habit all the time and thus making him/her anxious. Reduced bulk of bead does not obstruct while eating, presents minimum disturbances with speech, and stimulates tongue movement. It is esthetic and child becomes comfortable quickly. The patient believes to have acquired a new toy in mouth to play with tongue. 

On the other side, direct relationship between age and time of appliance placement has been observed. The younger the patients are, the more quickly and completely the tongue position becomes normalized and the lesser the time required for cessation of the habit is. Limbrock et al. [20] suggested the appliance design even for toddler group to 12 year old child. Cessation of habit was reported on very 1st day in toddlers, whereas it takes few weeks in case of 10–12-year-old children. Hence, in early mixed detention or even in younger group, appliance could be used comfortably. If habit persists for longer time exhibiting posterior cross bite, modified blue grass appliance can be given with Quad Helix [21] to expand arch. Hence, two stage treatments can be completed with single appliance with correction of habit.

## 4. Conclusion

Hence, modified blue grass appliance is a non punitive appliance and esthetic and child can wear it comfortably. It can be given as a supportive therapy as it requires no reminding or bribing, and parents can be freed of anxiety and frustration. It does not interfere with child's growth and eliminates the habit with limited complications.
